# Changes in Group B *Streptococcus* Colonization among Pregnant Women before and after the Onset of the COVID-19 Pandemic in Brazil

**DOI:** 10.3390/pathogens11101104

**Published:** 2022-09-27

**Authors:** Natália Silva Costa, André Rio-Tinto, Isabella Bittencourt Ferreira Pinto, Danielle Cristina dos Santos Silva Alvim, Amanda de Assis Rocha, Laura Maria Andrade Oliveira, Ana Caroline Nunes Botelho, Sergio Eduardo Longo Fracalanzza, Lucia Martins Teixeira, Jorge Rezende-Filho, Penélope Saldanha Marinho, Joffre Amim Júnior, Stephen Taylor, Steve Thomas, Tatiana Castro Abreu Pinto

**Affiliations:** 1 Departamento de Microbiologia Médica, Instituto de Microbiologia Paulo de Góes, Universidade Federal do Rio de Janeiro, Rio de Janeiro 21941-902, Brazil; 2 Faculdade de Medicina, Maternidade Escola, Universidade Federal do Rio de Janeiro, Rio de Janeiro 22240-000, Brazil; 3 UK Health Security Agency, Porton Down, Salisbury SP4 0JG, UK

**Keywords:** *Streptococcus agalactiae*, group B *Streptococcus*, pregnant women, anovaginal colonization, antimicrobial resistance, COVID-19 pandemic

## Abstract

Group B *Streptococcus* (GBS) is a leading cause of neonatal infections. The genitourinary and gastrointestinal tract of pregnant women are the main source of transmission to newborns. This work investigated the prevalence and characterized GBS from pregnant women in Rio de Janeiro, Brazil, comparing the periods before (January 2019 to March 2020; 521) and during (May 2020 to March 2021; 285) the COVID-19 pandemic. GBS was detected in 10.8% of anovaginal samples. Considering scenarios before and during the pandemic, GBS colonization rate significantly decreased (13.8% vs. 5.3%; *p* = 0.0001). No clinical and sociodemographic aspect was associated with GBS carriage (*p* > 0.05). A total of 80%, 13.8% and 4.6% GBS strains were non-susceptible to tetracycline, erythromycin and clindamycin, respectively. Serotype Ia was the most frequent (47.7%), followed by V (23.1%), II (18.4%), III (7.7%) and Ib (3.1%). An increasing trend of serotypes Ib and V, as well as of antimicrobial resistance rates, and a decreasing trend of serotypes II and III, were observed after the pandemic onset, albeit not statistically significant (*p* > 0.05). The reduction in GBS colonization rates and alterations in GBS serotypes and resistance profiles during the pandemic were not due to changes in the sociodemographic profile of the population. Considering that control and preventive measures related to the COVID-19 pandemic onset have impacted other infectious diseases, these results shed light on the need for the continuous surveillance of GBS among pregnant women in the post-pandemic era.

## 1. Introduction

*Streptococcus agalactiae* (Group B *Streptococcus*, GBS) is a leading cause of neonatal invasive diseases, alongside sepsis and meningitis, with mortality rates up to 50% [1]. Vertical transmission is the main route for newborn colonization, since this microorganism can be found in the genitourinary and gastrointestinal tract of pregnant women, with an estimated worldwide prevalence of 18%, ranging from 10 to 40% depending on the geographical region [1,2,3]. GBS has a polysaccharide capsule that allows the recognition of ten different serotypes (Ia, Ib, II-IX) and their distribution is also variable according to geographical region [4]. GBS capsule has been used as a basis for epidemiological and pathogenicity studies, and as the main targets for the development of GBS vaccines [5,6,7,8,9].

The US Centers for Disease Control and Prevention (CDC) recommends universal anovaginal screening of pregnant women between the 36th and 37th gestational weeks, submitting those who are colonized by GBS for intrapartum antibiotic prophylaxis (IAP). IAP is the only currently available prophylactic method against GBS neonatal infections, and despite preventing about 29,000 early onset disease cases, it has no impact on the incidence of prenatal-onset and late-onset disease [2,10,11]. Furthermore, universal screening and IAP are routinely applied mostly in high-income countries (HIC) but are not widely accessible to pregnant women in many low- and middle-income countries (LMIC), which bear the highest burden of GBS disease [1]. In Brazil, although there is no national policy for GBS screening or prophylaxis in pregnant women, the Brazilian Society for Pediatrics has reiterated CDC recommendations since 2011 [12].

Penicillin is the first choice for IAP, and although GBS is still recognized as universally susceptible to this antibiotic, strains with reduced susceptibility to beta-lactams have been sporadically reported since 2008, with rates around 2% [13,14,15,16,17]. Clindamycin is the second choice for pregnant women allergic to penicillin at high risk of anaphylaxis. However, high clindamycin and erythromycin resistance rates have been reported in different countries [18,19], leading to the inclusion of GBS in the CDC’s list of antibiotic resistance threats [20]. 

Since the onset of the COVID-19 pandemic, it is estimated that more than 18.2 million people died worldwide because of the disease [21]. In Brazil, the impact of COVID-19 on pregnant women was markedly high and associated with increasing mortality rates, from 7.4% in 2020 to 15.6% in 2021 [22,23]. Numerous infection control and preventive measures were implemented to mitigate the effects of the pandemic, including social distancing, mask wearing, practicing good hand hygiene and quarantine. These measures had an unprecedented impact in different aspects of society and public health and have led to the reduction in the incidence of other respiratory infectious diseases such as pneumococcal pneumonia [24,25,26,27].

On the other hand, the pandemic has revealed that increased pressure on healthcare systems can lead to abuse and/or misuse of antibiotics and deprioritization of antimicrobial resistance (AMR) surveillance. Higher frequencies of broad-spectrum antibiotics usage have been reported in certain places when compared with the pre-pandemic period [28]. Moreover, the generalized fear of COVID-19 disease along with the lack of public knowledge on how antibiotics work has led to increased access to over-the-counter antibiotics and consequent self-medication, especially in certain LMIC where control of antibiotic sale is still inadequate. In Peru, self-medication before hospital admission was reported by 33% of people during the pandemic [29,30,31].

Changes in societal behavior and clinical practices related to the pandemic might have simultaneously led to a reduction in the transmission of certain infectious diseases and to the selection for AMR, drawing attention to consequences beyond those directly linked to COVID-19. Thus, this work aimed to evaluate the prevalence and characteristics of GBS circulating in pregnant women in Rio de Janeiro, Brazil, between the periods before and after the COVID-19 pandemic onset. 

## 2. Materials and Methods

### 2.1. Study Population and Collection of Anovaginal Samples

A total of 806 anovaginal specimens were obtained from pregnant women attending the Teaching Maternity of UFRJ, in Brazil, between the 35th and 37th gestational weeks during routine antenatal care, from January 2019 to March 2021. The project was approved by the local research ethics committee under number 43389321.9.0000.5257 and written informed consent was obtained from all participants. Additionally, clinical and sociodemographic aspects were collected through a questionnaire and data were analyzed. The questionnaire included information such as age range, region of birth, marital status, ethnicity, level of education, previous preterm delivery, previous prenatal death, previous neonatal GBS infection, vaginal discharge, history of urinary tract infection and use of antibiotics, and pre-existing pathologies.

Anovaginal specimens were collected using the combined swab method, according to the American Society for Microbiology recommendations [32], where a single swab is obtained from each participant. Briefly, flocked swabs (Copan Diagnostics Inc., Murrieta, CA, USA) were first introduced in the middle third of the vaginal region, then introduced in the anus through the anal sphincter and were maintained in STGG (skim milk, tryptone, glucose, and glycerin) transport media and immediately forwarded to our laboratory. Clinical samples were collected continuously throughout the period analyzed, except for April 2020, which marked the beginning of the COVID-19 lockdown, and therefore no samples were collected during this month. Clinical specimens from January 2019 to March 2020 were considered representatives of the pre-pandemic period (n = 521), while specimens collected from May 2020 to March 2021 were considered representatives of after the onset of the COVID-19 pandemic (n = 285).

### 2.2. Detection and Isolation of GBS 

Aliquots of the STGG medium containing the swabs were subjected to a pre-enrichment step in Todd-Hewitt broth (Sigma-Aldrich, St. Louis, MO, USA) supplemented with nalidixic acid (15 μg/mL; Sigma-Aldrich, USA) and gentamicin (8 μg/mL; Sigma-Aldrich) as previously described [33]. After incubation for 24 hours at 37 °C, cultures were streaked onto chromogenic media (CHROMagar™ StrepB, CHROMagar, Paris, France) and the identification of colonies with a mauve appearance was performed by MALDI-TOF MS (Bruker Microflex LT, Bruker Daltonics, Bremen, Germany), according to manufacturer’s instructions.

### 2.3. Characterization of GBS Strains

Capsular typing was performed by using the commercial latex agglutination test Immulex Strep-B (SSI Diagnostica, Hillerod, Dinamarca), according to the manufacturer’s instructions. Antimicrobial susceptibility to penicillin, erythromycin, clindamycin, levofloxacin, tetracycline, and vancomycin (Oxoid, Basingstoke, United Kingdom) was determined by disk-diffusion according to CLSI guidelines [34]. 

### 2.4. Statistical Analysis

Statistical analysis was performed using two-way ANOVA and Fisher’s exact test with the support of GraphPad Prism software version 9.3.1 (GraphPad Software, La Jolla, CA, USA); *p*-values of <0.05 were considered statistically significant.

## 3. Results 

According to the questionnaire answered by the pregnant women enrolled in the study, the main sociodemographic profile was 29.5-year-old, born in the Southeast region, single, self-declared white, with high school level of education (Table 1). Regarding clinical data, 20–30% reported vaginal discharge, urinary tract infection and/or use of antibiotics during pregnancy (Table 1). Up to 10% declared to have had previous preterm delivery, previous neonatal death or previous neonatal GBS infection, while nearly 60% reported a pre-existing pathology (Table 1). The most frequently used antibiotics in this group were cephalexin (10.3%), followed by nitrofurantoin (5.1%), and amoxicillin (4%), while the most common pre-existing pathologies were diabetes (34.1%), arterial hypertension (15.8%), hypothyroidism (7.2%) and obesity (1.8%). Comparison between the sociodemographic and clinical data obtained in the periods before and during the COVID-19 pandemic did not show statistical significance (*p* > 0.05) (Table 1).

GBS was detected in 10.8% (87/806) of anovaginal samples. Considering the scenarios before and after the onset of COVID-19, GBS colonization rate significantly decreased after pandemic onset (13.8% versus 5.3%; *p* = 0.0001). In addition, sociodemographic and clinical data according to the presence or absence of GBS showed no significant difference (*p* > 0.05) (Table 2).

**Figure 1:**
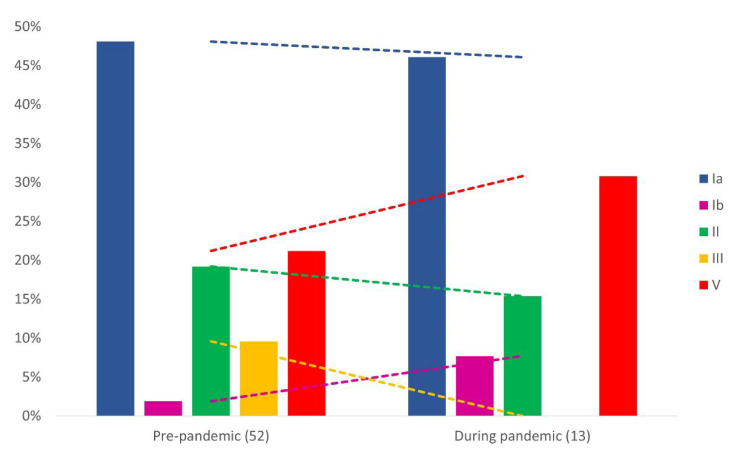
Distribution of serotypes among 65 group B *Streptococcus* strains recovered from pregnant women in Rio de Janeiro, Brazil before (pre-pandemic) and after the onset of COVID-19 (during pandemic). Dotted lines represent the tendency lines.

Although 87 GBS strains were recovered during the study, only 65 isolates remained viable to be submitted to additional characterization, including 52 strains from pre- and 13 from post-COVID-19 onset. Among these 65 GBS strains, serotype Ia (47.7%; 31) was the most frequent, followed by serotypes V (23.1%; 15), II (18.4%; 12), III (7.7%; 5) and Ib (3.1%; 2). When analyzing the two different scenarios (pre- and post-COVID-19 onset) separately, serotype Ia remains as the most common in both, being represented by nearly half of GBS strains before and during pandemic (48.1% and 46.1%, respectively). On the other hand, despite not being statistically significant, an increasing trend of serotypes Ib (1.9% vs. 7.7%) and V (21.2% vs. 30.8%) was detected after the onset of COVID-19, while serotype II decreased (19.2% vs. 15.4%) and serotype III was not detected during the pandemic (9.6% vs. 0%) (Figure 1). 

**Figure 2:**
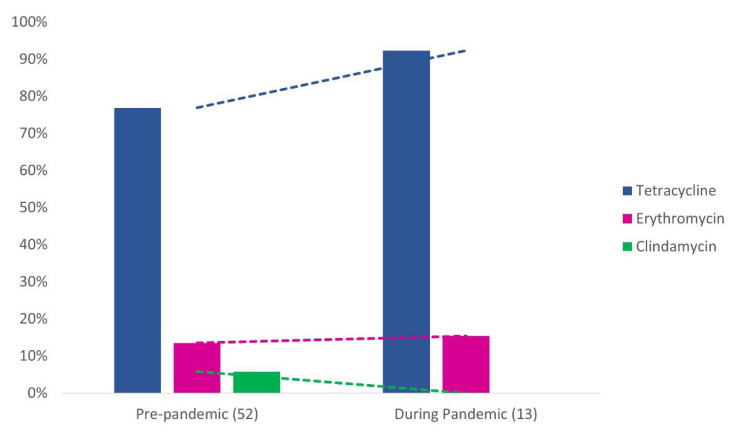
Antimicrobial resistance rates among 65 group B *Streptococcus* strains recovered from pregnant women in Rio de Janeiro, Brazil before (pre-pandemic) and after the onset of COVID-19 (during pandemic). Dotted lines represent the tendency lines.

All 65 GBS strains evaluated were susceptible to penicillin, vancomycin, and levofloxacin according to the CLSI guidelines [34], while 80%, 13.8% and 4.6% of strains were non-susceptible to tetracycline, erythromycin, and clindamycin, respectively. No significant difference (*p* > 0.05) in GBS susceptibility rates was detected between pre- and post-onset of the pandemic. However, clindamycin resistance was not detected during the pandemic and an increasing trend in erythromycin (13.5% vs. 15.4%) and tetracycline (76.9% vs. 92.3%) resistance was observed (Figure 2). 

In general, serotypes Ib, II and V harbored the highest proportion of strains non-susceptible to any of the antimicrobial agents tested. When comparing the two scenarios (pre- and post-COVID-19 onset), the proportion of antimicrobial non-susceptible GBS strains increased during the pandemic within serotypes Ia, II and V (Table 3).

## 4. Discussion 

Group B *Streptococcus* is a major cause of invasive infections in neonates, with the colonization of the anovaginal tract of pregnant women being the main transmission source. However, especially in LMIC such as Brazil, GBS is often neglected during antenatal care. Although there is no national policy for GBS screening and IAP established by the Brazilian Ministry of Health, the Brazilian Society for Pediatrics has recommended culture-based GBS screening since 2011 [12], but adhesion to these guidelines still seems to be low in Brazil [35]. Specifically in Rio de Janeiro, the secretary of health does not recommend universal GBS screening among pregnant women, based on the argument that there is no solid evidence of the burden of GBS in our setting. However, the city registered neonatal and perinatal mortality rates of 8.2/1000 and 15.4/1000 live births, respectively, between 2019 and 2021. Although the exact etiology of these deaths is not well defined, it is known that bacterial sepsis and pneumonia occupy the highest positions in the rank of childhood mortality in Brazil [36]. 

The overall GBS colonization rate detected in this study was 10.8%, a lower rate compared to other studies conducted in Brazil. A previous study performed by our group, also in Rio de Janeiro, evaluated pregnant women during an 8-year period and observed a colonization rate of 22–32% [33]. Other previous studies reported rates ranging from 17.4% to 27.6% in the Southeast region of Brazil [37,38,39,40], and of 28.4% in the South region of the country [41]. In a meta-analysis study conducted by Kwatra and colleagues [42], the worldwide estimated prevalence of maternal GBS colonization was 17.9%, with the highest estimated rate in Africa (22.4%), followed by the Americas (19.7%) and Europe (19%), and southeast Asia showing the lowest estimated prevalence (11.1%). 

However, GBS incidence rates can significantly vary, not only according to geographical region but also to period of time. When comparing pre- and post-COVID-19 onset scenarios, we detected a remarkable decrease in GBS maternal colonization in Rio de Janeiro, Brazil, from 13.8% to 5.3%. No statistical difference was seen regarding clinical and sociodemographic data between the two scenarios, indicating that this significant drop in GBS incidence was not related to modifications in the profile of the target population. Measures implemented to mitigate the COVID-19 pandemic, such as those related to hygiene practices, social distancing or the use of antiseptics and antibiotics, have contributed to alterations in the dynamics of certain infectious diseases [24,25,26,27], and thus may have also contributed, at least in part, to modifications in GBS occurrence in anovaginal microbiota of the population analyzed.

Although not statistically significant, changes in serotypes’ distribution were observed with the increase in serotypes Ib and V and the decrease in serotypes II and III during the pandemic. However, the overall prevalence of serotype Ia, followed by V, II, III, and Ib, was predominantly maintained when the two periods were analyzed separately. The exception was serotype Ib, which was more frequent than serotype III after the onset of COVID-19. Serotype distribution may also vary according to geographical region. Serotype Ia, the most predominant in this study, is indeed one of the most prevalent serotypes in the entire world. Maternal colonization by serotype Ia is globally reported, being the most predominant in maternal disease. Serotype Ia is also prevalent in early onset disease (EOD), especially in South America where it is more frequent than serotype III in EOD cases [43]. A meta-analysis study with GBS strains colonizing pregnant women from Africa revealed that serotype V was the most common, followed by III, Ia, Ib and II [44]. In Italy, the high prevalence of serotype III, followed by serotypes V, Ia, Ib, II, and IV [45] was recently reported. Studies in America have reported a high prevalence of serotypes Ia, Ib, II, III, IV, and V, with variations depending on the country, except for serotype Ia, which has been detected as the most frequent in this setting. In a recent study in the USA, serotype Ia was the most frequently detected, followed by V, II, III, Ib and IV [46]. A previous study of our group has reported the high prevalence of serotype Ia, followed by serotypes II, Ib, V, III and IV [33]. More recently, also in the Southeast region of Brazil, serotype Ia, followed by V, II, III, Ib, IV and VIII were detected among pregnant women [47]. Thus, serotype distribution detected in this study agrees with what has been reported in other studies on the American continent. Although studies from Africa and Asia frequently report the prevalence of more recently discovered serotypes VI, VII, VIII and IX [48,49], none of these were detected in the present study. 

IAP is the current prevention measure against neonatal infections caused by GBS. Despite being effective, the use of antibiotics in the antenatal period has generated a growing global debate about its implications, since it contributes to the selection of antibiotic-resistant bacteria and leads to questions about the validity of using these drugs as a prophylactic measure against GBS infections. Moreover, GBS was recently included in the CDC’s list of current antibiotic resistance threats [20] due to an increased detection of erythromycin and clindamycin resistant GBS strains. Rates of over 50% for erythromycin and 40% for clindamycin in the USA, and of over 60% for both antimicrobial agents in Taiwan and China [18,19,50] have been reported. However, in Brazil, resistance to erythromycin and clindamycin is still reported in the minority of strains tested, showing a decreasing trend in the last decade [33,51,52]. In the present study, rates of non-susceptibility to erythromycin (13.8%) and clindamycin (4.6%) were similar to studies carried out previously in Brazil that report rates around 14% for erythromycin and 2% for clindamycin [33,53]. Moreover, in this study, non-susceptibility to tetracycline was a common phenotype in GBS strains (80%), being also similar to that reported previously, with resistance rates between 81.7% and 97% [33,51,52].

Although no significant difference was detected in GBS susceptibility rates between before and after the onset of COVID-19, an increasing trend in erythromycin and tetracycline resistance rates was observed during the pandemic, which highlights the importance of keeping track of antimicrobial resistant GBS strains in our setting. It is noteworthy to mention that macrolides were the second most prescribed drug for COVID-19 in many places of the world, including Brazil, which may push even further the emergence of erythromycin resistance among bacteria [31]. Furthermore, although antimicrobial resistant strains were detected in all serotypes in this study, the increasing trend of serotype V during the pandemic deserves close monitoring, since this serotype has been historically associated with the emergence of macrolide resistance in GBS [54]. 

No significant statistical difference was detected among clinical and sociodemographic aspects evaluated between GBS-positive and GBS-negative women. Similarly, in Jordan, no clinical or demographic factor could be associated with GBS colonization in pregnant women [55]. However, other studies have reported that certain clinical and sociodemographic aspects, such as urinary tract infection [41] and vaginal discharge [33] in Brazil, and black ethnicity [56] in the United States, can be associated with increased risk of GBS carriage.

The low prevalence of GBS in the target population of this study implies a low number of GBS strains to be recovered and analyzed. Additionally, GBS screening of pregnant women is challenging in Brazil once this is not a practice widely performed in healthcare facilities across the country, making it difficult to investigate and analyze anovaginal specimens of pregnant women from multiple regions at the same time. The results of the present study represent observations of the impact of COVID-19 among pregnant women in Rio de Janeiro, Brazil, and need further and continuous evaluation in the following years, besides expanding similar studies to other regions of the world, to achieve more robust conclusions.

Studies that can contribute with data to develop and improve public health policies and epidemiological surveillance, mainly in the post-pandemic world, are of paramount importance. We have shown a remarkable reduction in GBS anovaginal colonization among pregnant women in Rio de Janeiro, Brazil after the onset of COVID-19. Furthermore, despite not being statistically significant, we have detected changes in certain characteristics of GBS strains isolated before and during the pandemic, including an increasing trend in antimicrobial resistance rates and variations in the distribution of GBS serotypes. Such observations were not due to changes in population profile nor in the methodology for GBS detection, suggesting they may be the result of natural oscillation or still unknown factors. Although control and preventive measures related to the COVID-19 pandemic onset may have impacted in other infectious diseases, we still do not know if this would also be true for GBS colonization in pregnant women. Therefore, our results draw attention to the importance and need for the continuous surveillance of GBS in the post-pandemic era.

## Figures and Tables

**Table 1:** Clinical and sociodemographic aspects of the pregnant women included in the study according to time period.

Aspects Evaluated	All (19 January to 21 March) n = 806	Before COVID-19 (19 January to 20 March) n = 521	After Onset of COVID-19 (20 May to 21 March) n = 285	*p*-Value
**Sociodemographic ^a^**				
Mean age	29.5 (13–46 years old)	29.6 (13–46 years old)	29.4 (13–45 years old)	0.8897
Region of birth ^b^				
Southeast	77.7% (580/746)	74.5% (361/485)	83.9% (219/261)	0.9978
North	0.8% (6/746)	1% (5/485)	0.4% (1/261)	
Northeast	19.8 % (148/746)	23.1% (112/485)	13.8% (36/261)	
Midwest	0.3% (2/746)	0.2% (1/485)	0.4% (1/261)	
South	0.7% (5/746)	0.8% (4/485)	0.4% (1/261)	
Marital status				
Married	47.9% (346/722)	46.4% (219/472)	50.8% (127/250)	1.0000
Single	52.1 (376/722)	53.6% (253/472)	49.2% (123/250)	
Ethnicity ^c^				
Black	20.6% (158/766)	20.2% (99/491)	21.5% (59/275)	1.0000
Brown	37.9% (290/766)	40.3% (198/491)	33.5% (92/275)	
White	40.9% (313/766)	38.9% (191/491)	44.4% (122/275)	
Level of education ^d^				
Basic education	23.1% (163/707)	26% (120/461)	17.5% (43/246)	0.9998
High school	59.5% (421/707)	58.6% (270/461)	61.4% (151/246)	
Higher education	17.3% (122/707)	15.2% (70/461)	21.1% (52/246)	
**Clinical ^a^**				
Previous preterm delivery	9.6% (74/773)	10.2% (51/498)	8.4% (23/275)	1.0000
Previous neonatal death	4.4% (33/757)	4.1% (20/486)	4.8% (13/271)	1.0000
Previous neonatal GBS infection	0.4% (3/717)	0.4% (2/461)	0.4% (1/256)	1.0000
Vaginal discharge	27.5% (200/728)	27.3% (129/472)	27.7% (71/256)	1.0000
Urinary tract infection	23.4% (180/770)	23.6% (118/500)	23% (62/270)	1.0000
Use of antibiotics	28.8% (218/757)	29.2% (144/493)	28% (74/264)	1.0000
Pre-existing pathologies	55.7% (427/766)	58.8% (291/495)	50.2% (136/271)	1.0000

^a^ Statistical analyses were performed using two-way ANOVA test; ^b^ Ten participants were from foreign countries; ^c^ Five participants self-declared as Indigenous or Asian; ^d^ One participant self-declared as illiterate. Numbers in parenthesis show the number of positive answers/number of patients who answered the question.

**Table 2:** Clinical and sociodemographic aspects of the pregnant women included in the study according to the presence or absence of group B *Streptococcus* (GBS) in the anovaginal samples.

Aspects Evaluated	All Clinical Samples n = 806	GBS Positive Samples n = 87	GBS Negative Samples n = 719	*p*-Value
**Sociodemographic ^a^**				
Mean age	29.5 (13–46 years old)	29.8 (13–43 years old)	29.5 (13–46 years old)	0.9292
Region of birth ^b^				
Southeast	77.7% (580/746)	72.8% (59/81)	78.3% (521/665)	0.9999
North	0.8% (6/746)	0% (0/81)	0.9% (6/665)	
Northeast	19.8 % (148/746)	26% (21/81)	19.1% (127/665)	
Midwest	0.3% (2/746)	0% (0/81)	0.3% (2/665)	
South	0.7% (5/746)	0% (0/81)	0.7% (5/665)	
Marital status				
Married	47.9% (346/722)	43.2% (35/81)	45% (311/691)	0.6662
Single	52.1 (376/722)	56.8% (46/81)	47.8% (330/691)	
Ethnicity ^c^				
Black	20.6% (158/766)	24.1% (20/83)	20.2% (138/683)	0.9920
Brown	37.9% (290/766)	33.7% (28/83)	38.4% (262/683)	
White	40.9% (313/766)	42.2% (35/83)	40.7% (278/683)	
Level of education ^d^				
Basic education	23.1% (163/707)	32.4% (24/74)	22% (139/633)	0.9948
High school	59.5% (421/707)	48.6% (36/74)	60.8% (385/633)	
Higher education	17.3% (122/707)	17.6% (13/74)	17.2% (109/633)	
**Clinical ^a^**				
Previous preterm delivery	9.6% (74/773)	9.5% (8/84)	9.6% (66/689)	1.0000
Previous prenatal death	4.4% (33/757)	2.4% (2/82)	4.6% (31/675)	1.0000
Previous neonatal GBS infection	0.4% (3/717)	0% (0/77)	0.5% (3/640)	1.0000
Vaginal discharge	27.5% (200/728)	30.5% (25/82)	27.1% (175/646)	1.0000
Urinary tract infection	23.4% (180/770)	17.9% (15/84)	24% (165/686)	1.0000
Use of antibiotics	28.8% (218/757)	20.5% (17/83)	29.8% (201/674)	1.0000
Pre-existing pathologies	55.7% (427/766)	56.1% (46/82)	55.7% (381/684)	1.0000

^a^ Statistical analyses were performed using two-way ANOVA test; ^b^ Ten participants were from foreign countries; ^c^ Five participants self-declared as Indigenous or Asian; ^d^ One participant self-declared as illiterate. Numbers in parenthesis show the number of positive answers/number of patients who answered the question

**Table 3:** Distribution of antimicrobial non-susceptible (NS) group B *Streptococcus* (GBS) strains recovered from pregnant women in Rio de Janeiro, Brazil, according to serotype and time period.

Serotype	All NS ^a^ Strains (19 January to 21 March) n = 52	NS Strains before COVID-19 (19 January to 20 March) n = 40	NS Strains after Onset of COVID-19 (20 May to 21 March) n = 12	*p*-Value ^b^
Ia	77.4% (24/31)	76% (19/25)	83.3% (5/6)	1.0000
Ib	100% (2/2)	100% (1/1)	100% (1/1)	N/A ^c^
II	91.7% (11/12)	90% (9/10)	100% (2/2)	1.0000
III	60% (3/5)	60% (3/5)	0% (0/0)	N/A^c^
V	80% (12/15)	72.7% (8/11)	100% (4/4)	0.5165

^a^ NS: Non-susceptible to any of antimicrobial agents tested; ^b^ Statistical analyses were performed using Fisher’s exact test; ^c^ N/A: Not applicable. Numbers in parenthesis show the number of positive answers/number of patients who answered the question.

## Data Availability

The data presented in this study are available in the present article “Changes in Group B *Streptococcus* Colonization among Pregnant Women before and after the Onset of the COVID-19 Pandemic in Brazil” or available on request from the corresponding author.
